# Challenges for setting up psychiatric services in a trauma centre in India

**DOI:** 10.1192/bji.2023.35

**Published:** 2024-02

**Authors:** Nishtha Chawla, Rakesh K. Chadda

**Affiliations:** 1 Assistant Professor, Department of Psychiatry, All India Institute of Medical Sciences, Delhi, India; 2 Professor and Head, Department of Psychiatry, All India Institute of Medical Sciences, Delhi, India. Email: drrakeshchadda@gmail.com

**Keywords:** Trauma and stressor-related disorders, consultation-liaison psychiatry, mental health services, trauma centre, challenges

## Abstract

Psychiatric sequelae may occur following traumatic injury irrespective of whether an insult has been caused to the brain. A range of psychiatric illnesses have been either causative of or associated with road traffic accidents and traumatic injuries, including depression, anxiety, post-traumatic stress disorder, substance use disorder and attention-deficit hyperactivity disorder. Despite literature on such associations, psychiatric intervention in the treatment of patients following traumatic injury is limited. The authors share their experience of challenges in addressing mental health problems in a tertiary care trauma centre located in North India. Steps in overcoming those challenges included: developing a semi-structured form to be completed for referrals and consultations, a psychiatrist attending weekly rounds with the surgeons, and initiating a psychiatry out-patient clinic for patients discharged from the trauma centre. It may be worthwhile in the future to set up a trauma psychiatry unit at the centre, involving a clinical psychologist, a psychiatric social worker and an occupational therapist for the comprehensive care of patients.

Traumatic brain injury (TBI) often has behavioural and psychiatric sequelae, including delirium and a range of organic psychiatric disorders. Depression is common in TBI survivors, with a prevalence ranging from 18 to 61%, and mania has been found in nearly 4%.^1^ Obsessive–compulsive disorder is less common (affecting nearly 1%) but requires differentiation from other repetitive behaviours occurring due to perseveration.^1^ Other neuropsychiatric sequelae include post-traumatic stress disorder (PTSD), psychotic symptoms due to organic factors, and organic personality changes. In addition, psychiatric sequalae may occur without any direct brain insult during a traumatic event, due to either acute stress associated with the trauma or chronic stress associated with permanent disability occurring secondary to the traumatic event.

Disabling injuries such as amputation of a limb have a significant impact on the individual, especially when they occur as a result of sudden trauma.^2^ This leads to significant loss in function, sensation and body image, which directly affect psychological adjustment. In a sample of Jordanian in-patients and out-patients with unilateral lower limb amputation, 37% reported anxiety and 20% reported depressive symptoms.^2^

Attention-deficit hyperactivity disorder (ADHD) is an important psychiatric condition often discussed in association with road traffic accidents (RTAs). In a German study involving 905 accident victims admitted to hospital for trauma surgery, prevalence of ADHD was found to be 6.18% and most individuals had not received a previous diagnosis.^3^ In a study from Iran, individuals with ADHD were identified as having higher odds of risky driving and being involved in a car accident.^4^

Another condition which is not uncommon in individuals injured in RTAs is substance use. A study in Iran found a significant association between drug use and the incidence of RTAs.^5^ A systematic review from India demonstrated that a significant proportion of individuals who were injured or had died in an RTA had consumed alcohol before the accident.^6^

There is literature on the association of psychosocial problems and psychiatric illnesses with traumatic injuries. A range of psychiatric and psychosocial problems play a major role in the occurrence of trauma as well as in recovery and rehabilitation of patients following traumatic injury.

We share here our experience of addressing mental health problems in a tertiary care trauma centre located in North India.

## Initial experiences of a psychiatrist deployed in the trauma centre

The first author (N.C.) joined the faculty in psychiatry at Jai Prakash Narayan Apex Trauma Centre, a lead multidisciplinary tertiary care trauma centre in North India, in December 2022. The centre is affiliated with the All India Institute of Medical Sciences (AIIMS), New Delhi, a leading medical institute in the country. The centre, which was established in 2006, provides holistic trauma care services and functions as a referral centre and a centre for training and education. It has nearly 300 beds, with 50 intensive care unit (ICU) beds and 30 emergency beds. Out-patient services are restricted to follow-up patients admitted from the casualty department and no new patients are seen in the out-patient clinic. Patients needing in-patient care are admitted in three departments, viz. trauma surgery, orthopaedics and neurosurgery. The centre receives nearly 120 emergency patients a day (around 43 999 in April 2020–March 2021), and on average 18 patients per day are admitted to different in-patient services (6420 in April 2020–March 2021). Out of more than 5000 surgical procedures performed in the year, more than 90% involved major surgery, with nearly half of patients having major fractures and 10–15% requiring neurosurgical interventions (for head and spine injuries).

In 2022 (16 years after the centre first opened), a dedicated psychiatry faculty was appointed at the centre to provide continued psychiatric care. Although psychiatric services had been provided in centre since its inception, on the basis of referrals received by the consultation psychiatry team of the parent institute, there was scope to improve knowledge about psychiatric problems among surgeons at the centre, as well as patients and their family members. Patients with self-inflicted injuries or attempted suicide would often receive surgeons’ attention, but various other factors affecting mental health could be missed or misidentified. For example, excessive pain despite adequate analgesia could be due to underlying depression or anxiety, and body image distortions in patients with amputation/phantom limb sensations could be underrecognised.

Currently, the psychiatry team liaise with the surgical teams for detailed rounds once a week. This offers the psychiatry team opportunity to understand surgical elements of the patient's illness and improves the surgeons’ understanding of the psychological aspects. Weekly rounds with a psychiatry junior resident trainee entail detailed work-up of patients by the resident, followed by discussion with the psychiatry consultant (N.C.). A semi-structured form has been developed for completion in psychiatry referrals. This helps not only in ensuring a more structured evaluation of the patient but also better training of the residents of both specialties. In addition, a weekly psychiatry out-patient clinic has been initiated to follow up patients who require psychiatric intervention during their in-patient stay or who are found to be experiencing mental health problems during their follow-up with the primary treating team.

Such a setting is different from the usual consultation-liaison psychiatry setting in AIIMS because it ensures continuity of clinical care and helps in understanding the nuances of psychiatric issues associated with various traumatic injuries, including their long-term course. Further, while attending rounds with surgical specialties, it is easier to understand their perspective about the psychiatric problems and expectations of psychiatric intervention.

## Starting psychiatric services: challenges and changing scenarios

A major challenge faced was to start psychiatry out-patient services in the trauma centre. Although it was known that the follow-up patients would require out-patient psychiatric care, making an appointment for a standard psychiatric work-up proved to be a challenge. The surgical specialties seeing follow-up patients discharged from the wards would require only a brief interaction time for assessment (essentially a follow-up assessment). A mental state assessment, on the other hand, would often require longer interaction time with patients, especially those who had cognitive deficits. Staff also had to be made aware of the need to maintain separate confidential case records, since psychiatric assessment would often include sensitive personal information about the patients. However, after initiation of a specialised psychiatry clinic, the quality of care has improved in terms of continuing long-term follow-up of patients by a dedicated psychiatrist. Since patients receive follow-up for their physical and psychiatric problems within the same centre, it is likely to reduce stigma associated with attending a separate psychiatry out-patient setting, in addition to cutting down on the cost, time and effort, especially in the presence of physical disability. However, patients who need in-patient psychiatric care have to be transferred to the parent institute (AIIMS), as this is currently unavailable in the trauma centre.

Lack of other mental health resources, such as a clinical psychologist, psychiatric social worker and mental health nurse, is yet another challenge faced. Such a multidisciplinary mental health team would play a crucial role in trauma settings, since stress-related disorders are likely to be common. Moreover, neuropsychological assessment of individuals with TBI may be warranted to plan therapy and occupational rehabilitation. In addition to clinical services, a mental health team could play a crucial role in improving the trauma care team's mental health and communication skills. Given the limited mental health resources, developing a ‘first-aid’ intervention for delivery in acute crisis by non-mental health professionals and paramedical staff would help the surgical team to manage mild cases on a day-to-day basis.

**Fig. 1:**
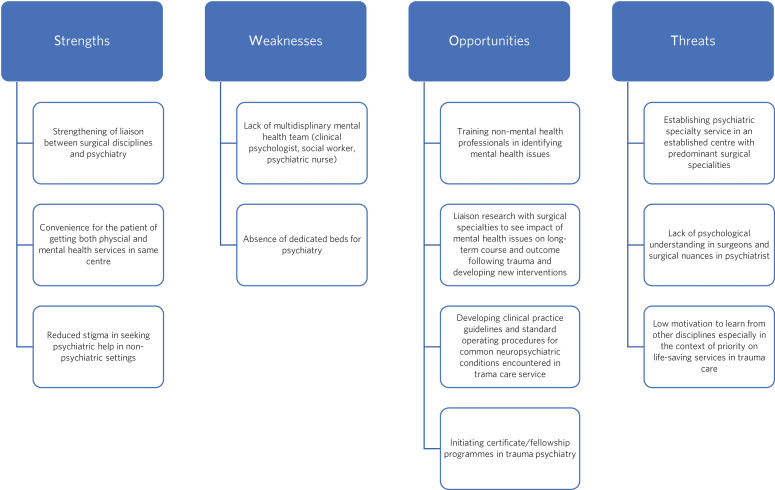
A strength–weakness–opportunity–threat (SWOT) analysis of setting up dedicated psychiatric services in Jai Prakash Narayan Apex Trauma Centre.

Dedicated psychiatric services in the trauma centre have also opened gates to focusing on research into mental health issues associated with RTAs, which are a major public health concern in India. Despite evidence on co-occurrence of psychiatric illness with various traumatic injuries and RTAs globally, there is scant published literature from India on the subject. Some literature is available on neuropsychiatric aspects of TBI. A study assessing the prevalence and correlates of aggression in TBI highlighted that 55% of patients admitted with TBI showed aggressive behaviour in acute care, and use of tobacco was an important association with this.^7^ A prospective Indian study on aggression and TBI highlighted that nearly one-third of patients showed aggressive behaviour at the time of admission and the functional outcome was dependent on the type and location of lesion, as well as the severity and duration of the aggression.^8^ Another prospective study in patients with TBI concluded that an initial Glasgow Coma Scale score of 10 may be crucial in predicting fewer cognitive abnormalities over a 6- to 12-month recovery period.^9^ In addition to cognition and aggression, some literature is available on affective symptoms among patients with TBI. A study conducted in an out-patient setting involving individuals post-TBI revealed that 37.7% reported mild depression and 34.4% reported moderate symptoms.^10^ In addition to severity of injury, various psychosocial factors, such as gender and unemployment, were significant predictors of depression. A case series of individuals with mild and moderate TBI observed minimal differences in cognitive tasks between the two types, with a higher propensity for developing affective symptoms in patients with mild TBI compared with the moderate group.^11^ A study involving patients who had had traumatic limb amputations found major depressive disorder to be the most common psychiatric comorbidity, present in over 70% of participants; suicidality was present in almost one-third of the sample. Presence of PTSD has been reported to be associated with phantom limb sensation/pain.^12^ With limited literature, small sample sizes, cross-sectional assessments and lower effect sizes observed in most studies, the conviction with which conclusions can be drawn from the current Indian literature is limited. Future studies should assess long-term outcome in terms of affective, cognitive and sensorimotor symptoms among patients who have suffered physical trauma and how these symptoms and their progression affect outcome and functioning. The role of culture and environment in determining outcome must also be studied. Studies targeting various intervention strategies may help adapt and validate evidence-based interventions in this population. Figure 1 shows a strength–weakness–opportunity–threat (SWOT) analysis of setting up a trauma psychiatry service in our trauma centre.

## New arenas in trauma psychiatry

Teaching surgeons and nurses to screen for psychiatric illness would strengthen their concept of mind–body interaction. Simple and short screening instruments such as the 7-item Generalised Anxiety Disorder assessment (GAD-7) and 9-item Patient Health Questionnaire (PHQ-9) in both the acute and rehabilitative phases of traumatic injury would provide useful information for clinical interventions. Involving a psychiatry resident in weekly rounds with the surgical team would be helpful in providing a two-way learning process for resident doctors in both disciplines. Early screening and treatment of psychiatric and psychosocial issues is likely to affect long-term outcomes. An important acute condition encountered in trauma centres is delirium. An important preventable cause of delirium is alcohol dependence, the prevalence of which is high in individuals involved in RTAs. By liaising with a psychiatrist, cases of impending delirium could be prevented or identified early and treated, which could potentially reduce the morbidity and mortality associated with it.

It may be worthwhile in future to form a trauma psychiatry unit and involve a clinical psychologist, a psychiatric social worker, psychiatric nurse and an occupational therapist for comprehensive care of patients.

Qualitative research is needed into relatively unexplored areas such as trauma patients’ need for psychiatric interventions. It is essential to incorporate psychological assessment and treatment into the rehabilitation of patients with physical disability due to trauma. Research pertaining to the development of clinical practice guidelines on the management of common psychiatric problems affecting individuals who have suffered physical trauma, for example aggression in those with TBI, and the development of standard operating procedures for cognitive and occupational rehabilitation and the assessment of fitness to return to work or provision of disability certification (where necessary) would be crucial steps in the future.

## Data Availability

Data availability is not applicable to this article as no new data were created or analysed in this study.
